# Beam-driven dynamics of aluminium dopants in graphene

**DOI:** 10.1088/2053-1583/ac6c30

**Published:** 2022-05-19

**Authors:** Georg Zagler, Maximilian Stecher, Alberto Trentino, Fabian Kraft, Cong Su, Andreas Postl, Manuel Längle, Nika Pesenhofer, Clemens Mangler, E Harriet Åhlgren, Alexander Markevich, Alex Zettl, Jani Kotakoski, Toma Susi, Kimmo Mustonen

**Affiliations:** 1 University of Vienna, Faculty of Physics, Boltzmanngasse 5, 1090, Austria; 2 Department of Physics, University of California, Berkeley, CA 94720, United States of America; 3 Materials Sciences Division, Lawrence Berkeley National Laboratory, Berkeley, CA 94720, United States of America; 4 Kavli Energy NanoSciences Institute at the University of California, Berkeley, CA 94720, United States of America; 5 Vienna Doctoral School in Physics, University of Vienna, Boltzmanngasse 5, Vienna 1090, Austria

**Keywords:** scanning transmission electron microscopy, atomic dynamics, heteroatom doping

## Abstract

Substituting heteroatoms into graphene can tune its properties for applications ranging from catalysis to spintronics. The further recent discovery that covalent impurities in graphene can be manipulated at atomic precision using a focused electron beam may open avenues towards sub-nanometer device architectures. However, the preparation of clean samples with a high density of dopants is still very challenging. Here, we report vacancy-mediated substitution of aluminium into laser-cleaned graphene, and without removal from our ultra-high vacuum apparatus, study their dynamics under 60 keV electron irradiation using aberration-corrected scanning transmission electron microscopy and spectroscopy. Three- and four-coordinated Al sites are identified, showing excellent agreement with *ab initio* predictions including binding energies and electron energy loss spectrum simulations. We show that the direct exchange of carbon and aluminium atoms predicted earlier occurs under electron irradiation, although unexpectedly it is less probable than the same process for silicon. We also observe a previously unknown nitrogen–aluminium exchange that occurs at Al–N double-dopant sites at graphene divacancies created by our plasma treatment.

## 1. Introduction

Heteroatom-substituted two-dimensional (2D) materials have generated sustained research interest [1]. Applications in fuel-cells, energy storage devices, sensing, catalysis [2–5] and not least, nanoelectronics [6, 7], have motivated numerous studies of these materials. Graphene with its outstanding electronic properties [8, 9], in particular its high electron mobility [10], stands out as a promising candidate for both smaller and more capable electronic devices [11, 12]. To this end, the electronic structure of monolayer graphene may need to be manipulated to open a bandgap, e.g. by lateral confinement [13, 14] or doping [7, 15, 16].

While graphene doping has been widely studied across the periodic table [17], with the period two neighbors to carbon, N and B, being the most commonly studied dopants [18–20], direct atomic-level evidence for the incorporation of other heteroatomic substitutions is more sparse. Si [21], P [22], Ge [23], O [24, 25], and Au [26] have been conclusively detected in monolayer graphene using atomically resolved electron energy loss spectroscopy (EELS) and/or quantitative image contrast comparisons with simulations. Several other elements, including many transition metals [3, 27, 28], have been identified on the basis of either non-local spectroscopy or chemically-insensitive imaging, although the identity of the substituted atoms was not in every case conclusively proven.

Beyond possible applications, research on dopants in graphene has produced insight into a rich variety of physical phenomena discovered by atomic-level observations. Heteroatoms in graphene are often found in multiple configurations, typically bonding either to three or four carbon neighbors [21, 29]. Aberration-corrected scanning transmission electron microscopy (STEM) can resolve their atomic configuration [30, 31], and additionally give detailed insight in the chemical structure with EELS [21, 22, 32]. Irradiation with the imaging electrons can, however, also induce dynamic structural changes in graphene [33, 34], including its heteroatom sites [35]. Indeed, utilizing the kinetic energy imparted by electrons, certain heteroatoms in graphene [29, 36, 37], single-walled carbon nanotubes (SWCNTs) [38], and within bulk silicon [39, 40] can be manipulated at atomic precision using the atomically focused STEM electron probe. This has opened new perspectives for engineering materials on the atomic level.

Period 3 elements Al, Si and P are all expected to be stable dopants in graphene [16], and to also show the richest dynamics under electron irradiation [29]. However, although there have been numerous theoretical studies on Al dopants [16, 41–43], their direct experimental detection has so far been limited to a single incidental example that was sputtered during EEL spectrum acquisition [29], and a very recent study using a chemical synthesis route [44]. However, characterization of the electron-beam stability of the Al heteroatom sites as well as their dynamics is still crucially missing, and a complementary physical post-synthesis modification route would be highly desirable.

Here, we report on Al heteroatom substitutions into graphene as well as on their electron-beam induced dynamics under 60 keV electron irradiation. We observe both three- and four-coordinated configurations whose atomically resolved EEL spectrum fine structure matches our first principles simulations. Notably, we first attempted to incorporate Al using low-energy ion implantation with Al^+^ energies around 30 eV, but despite exhaustive STEM characterization of multiple samples, could not locate any implanted impurities in the lattice. This was presumably due to excessive surface contamination that was either pre-existing on the surfaces, or was introduced in the merely high-vacuum implantation chamber and/or subsequent ambient transfer. Hence, in our view, direct low-energy ion implantation remains a highly challenging method. We therefore resorted to an intermittent vacancy approach to substitute Al into graphene, which has been used earlier to substitute heavy elements [26, 45], transition metals [28], and silicon [46, 47].

Commercial monolayer graphene supported on TEM grids was first irradiated by Ar ions to create vacancies, after which Al atoms were introduced by physical vapour deposition accompanied by laser heating, substituting them into some of the vacancies. STEM was used to image the dopants and observe their dynamics, and EELS to characterize their bonding. Supporting density functional theory (DFT) simulations were used to confirm the heteroatom bonding configurations and to reveal their three-dimensional structure and energetics: as expected, Al are observed in both three- and four-coordinated configurations, thus bonding to either three or four C neighbours.

As has been previously shown for Si and P heteroatoms [29, 48] in graphene and for Si also in carbon nanotubes [38], electron irradiation of Al dopant sites is expected [29] to result in the direct exchange of Al with one of its C neighbours, facilitating its migration within the graphene lattice without the loss of atoms. Slightly higher kinetic energy transfer from the electrons can lead to C atom ejection, converting a three-coordinated configuration into a four-coordinated one. The threshold energies for these processes for three-coordinated Al substitutions were previously estimated with DFT-based molecular dynamics (MD) simulations by Su *et al* [29], but the direct exchange mechanism was not experimentally confirmed until now. We also observed theoretically predicted [49] Al–N dual-doped configurations for the first time, and found that electron irradiation can also lead to their atomic rearrangement, whose mechanism we explain by DFT/MD simulations.

## 2. Results and discussion

### 2.1. Experimental

**Figure 1.**
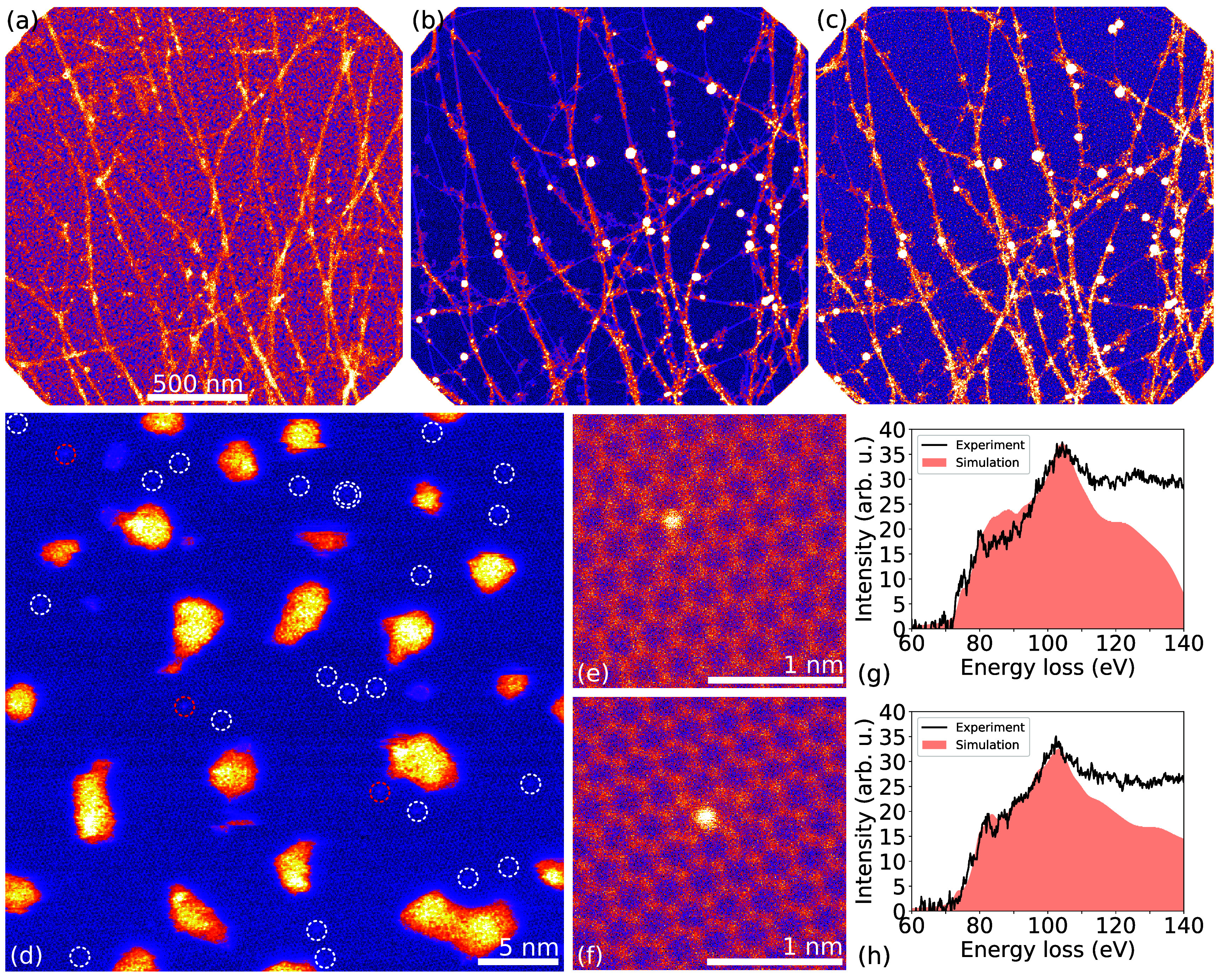
Al dopants substituted into suspended monolayer graphene (STEM/MAADF images and EEL spectra). (a) Overview of the specimen upon introduction to the vacuum system. Free-standing graphene and single-walled carbon nanotubes (SWCNTs, bright linear contrast) on the graphene are coated by hydrocarbon contamination (intermediate speckled areal contrast). (b) After heating with an *in-situ* laser, most contamination is removed from the graphene surface, with some contamination and heavier (bright spherical contrast) clusters remaining at the SWCNTs. (c) After ion irradiation and Al deposition, many small clusters cover the cleaned free-standing graphene (dense bright point contrast). (d) Closer view of the sample after cleaning, plasma irradiation and Al deposition. Bright contrast are nm-sized Al clusters formed after Al deposition. A total of 25 atomic sites (marked with dashed circles) in the graphene are substituted with heteroatoms. Of these, 22 sites (white) show a contrast consistent with either Al or Si heteroatoms, while three sites (red) appear clearly brighter than is expected for Al and are possibly Cu impurities remaining from the original graphene synthesis. (e) Close-up view of an Al heteroatom in a three-coordinated configuration. The corresponding EEL spectrum in (g) showing the Al $L_{2,3}$ core-loss edge (onset 73 eV). A simulated spectrum (solid filled light salmon colored area) shows good agreement with the experimental data (black line). (f) Close-up view image of an Al heteroatom in four-fold configuration. The corresponding EEL spectrum and simulation are shown in (h).

We prepared suspended graphene monolayers by placing commercially available chemical vapour deposition graphene onto perforated SiN grids, and then dry-deposited SWCNTs onto the graphene to improve mechanical stability [50]. After standard overnight bake, the samples were introduced into our interconnected ultra-high vacuum (UHV) system, where both heteroatom substitution and subsequent STEM and EELS characterization with the Nion UltraSTEM 100 instrument were performed. Hydrocarbon contamination covering the graphene surface (see figure 1(a)) was removed [51] using a high-power-density laser aimed inside the microscope column through a viewport. The resulting cleaned surface (see figure 1(b)) as well as sample transfers in near-UHV ensured that we could achieve large atomically clean areas [47]. For detail, see section 4.

The pre-cleaned sample was transferred to a plasma chamber within the UHV system and exposed to ca. 3 × 10^13^ cm^−2^ of Ar ions with a mean kinetic energy of ca. 170 eV, at which primarily mono- and divacancy defects are expected to form [52]. The sample was simultaneously irradiated with a laser to elevate its temperature and to mitigate hydrocarbon buildup. The plasma treatment was followed by thermal evaporation of an Al target heated to 955 ^∘^C, producing an Al partial pressure of 10^−8^ mbar. The total evaporation time was 20 s, resulting in the Al coverage observable in figures 1(c) and (d), where single dopants within the graphene lattice can be seen, as well as numerous Al nanoclusters. Although the heteroatom substitution yield was relatively high, roughly two thirds of the substituted heteroatoms were found to be Si instead, again highlighting its chemical affinity for graphene [47].

#### 2.1.1. Aluminium substitutions

As expected based on our own recent experiments on both graphene and SWCNTs [47, 53] and atomistic simulations of Ar irradiation of graphene conducted by others and reported in the literature [52], the Al dopants are mainly found in single and double vacancies, corresponding to three-(Al–C_3_) and four-(Al–C_4_) coordinated configurations [43] (figures 1(e) and (f)), similar to Si [21, 32], Ge [23], and P [22]. Both configurations were found in roughly equal numbers in the specimens, and their measured EEL spectra are in good agreement with the simulated ones (figures 1(g) and (h); section 4).

The experimentally determined projected Al–C distances were 1.65 ± 0.05 Å (Al–C_3_) and 1.96 ± 0.05 Å (Al–C_4_), being in an excellent agreement with the distances calculated from relaxed DFT models (section 4), which were 1.65 and 1.95 Å, corresponding to bond lengths of 1.86 and 1.96 Å, respectively. Notably, similar to the previously studied four-coordinated silicon impurity (Si–C_4_) [54], the Al–C_4_ ground state is slightly non-planar with the bonding C atoms displaced from the plane by ± 0.2 Å, showing signs of tetrahedral bonding [43]. The binding energies of the Al dopants into the sites were similar at −5.95 eV (Al–C_3_) and −4.76 eV (Al–C_4_). Stone-Wales-rotated configurations containing Al were not observed in our experimental data.

#### 2.1.2. Al–C bond inversion

**Figure 2.**
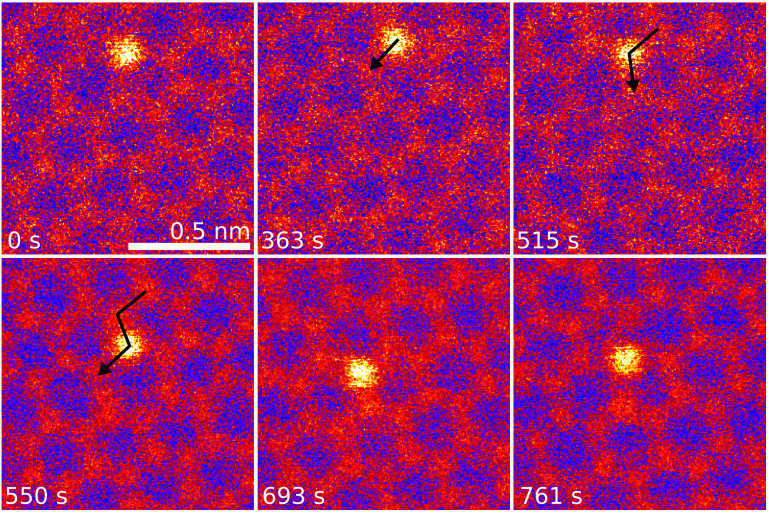
Three Al–C direct exchange steps both preceded and followed by conversion between four- and three-coordinated bonding. STEM/MAADF image frames selected each time a new configuration was observed. The overlaid numbers show the time elapsed since continuous recording of image series began. (**0** **s**) Al heteroatom in four-coordinated (Al–C_4_) configuration. (**363** **s**) The bonding is converted to Al–C_3_ by capture of a diffusing C adatom. (**515–693** **s**) The Al–C_3_ jumps three subsequent times from one lattice site to the next via direct exchanges with subsequent neighbouring C atoms. (**761** **s**) The bonding configuration is converted to Al–C_3_N by knock-out of a C neighbour; the atom downwards from the Al site is N (see figure 3 below).

**Figure 3.**
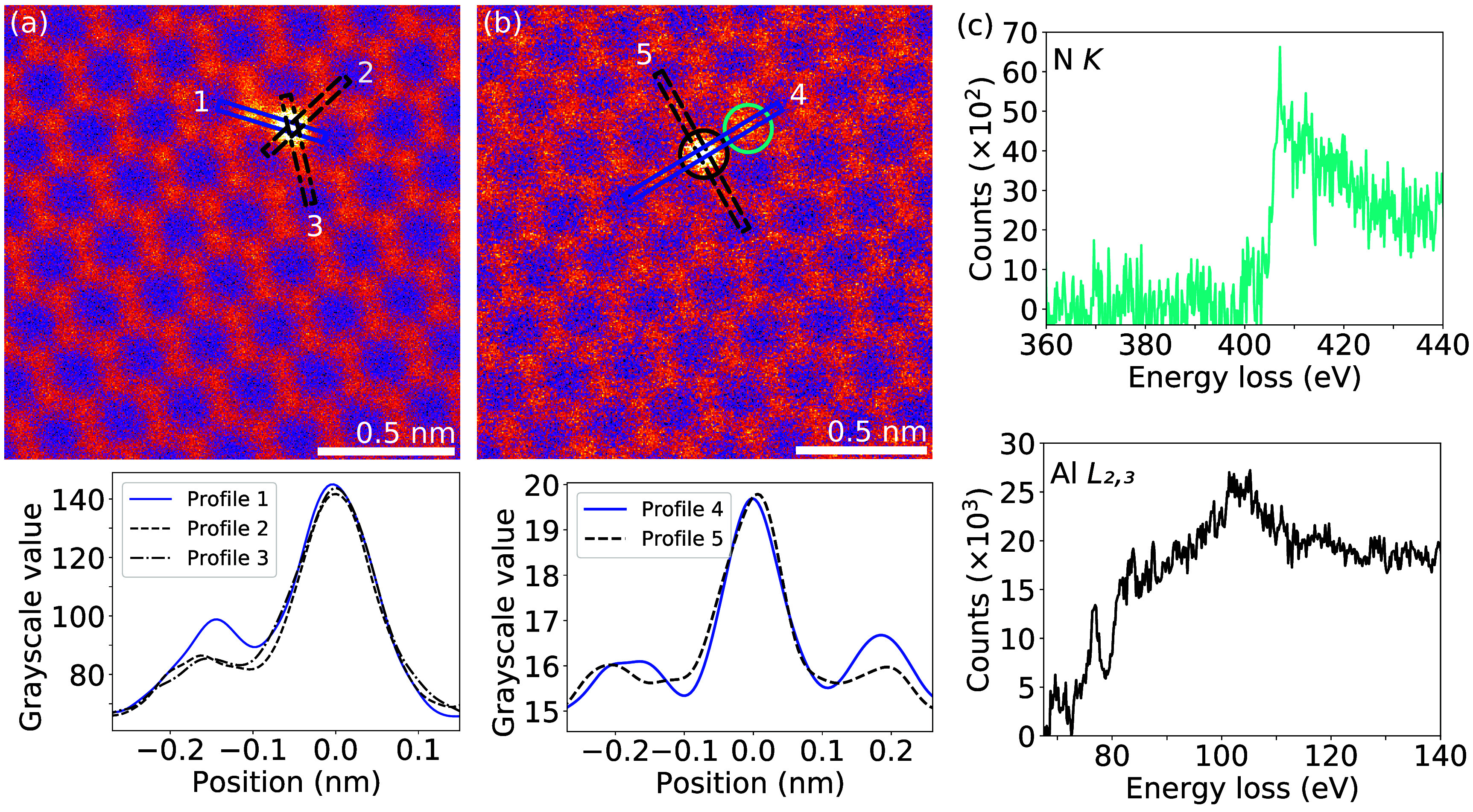
Al–N double-dopants in three-(Al–C_2_N) and four-coordinated (Al–C_3_N) configurations in the graphene lattice (STEM/MAADF images and EEL spectra). (a) Al bound to three neighbouring atoms. Line profiles of the intensity (along lines marked 1, 2 and 3) are plotted underneath after applying a low-pass filter. The atom on the top-left side of Al in profile 1 shows a higher intensity compared to the two other neighbors, consistent with a N heteroatom. (b) Al bound to four neighbouring atoms. Line profiles of the intensity (along lines marked 4 and 5) are plotted underneath after applying a low-pass filter. The atom on the top-right side of Al in profile 4 shows a higher intensity than the other neighbors, again consistent with a N heteroatom. (c) EEL spectrum (turquoise line) of the N atom encircled in turquoise in (b) with characteristic N *K* core-loss edge onset at ca. 400 eV. EEL spectrum (black line) of the central Al atom encircled in black in (b), showing an Al $L_{2,3}$ core-loss edge onset at 73 eV.

Al dopants exhibit various dynamic processes induced by the 60 keV electron irradiation. In figure 2 after 363 s of continuous observation, an Al heteroatom was converted from a four-coordinated Al–C_4_ to a three-coordinated Al–C_3_ configuration via the capture of a C adatom into the lattice [48]. In the subsequent frames (363–693 s), the direct exchange (bond inversion) of C and Al was observed three times prior to conversion into Al–C_3_-N with neighbouring a N heteroatom (see figure 3 below). For Si and P heteroatoms, migration and controlled manipulation has been demonstrated at the same electron energy [29, 34, 46]: carbon neighbours of these heteroatoms can swap atomic position with them. In this dynamical beam-induced process, an elastic momentum transfer from a single probe electron to a C atom causes it to nearly eject from the lattice, but during its upwards trajectory, the heteroatom relaxes into the created transient vacancy and the ejected atom is recaptured into the lattice at the heteroatom’s original position [48].

In a more recent study [29], direct exchange was also predicted for Al–C_3_ at 60 keV, with a notably lower range of threshold energies (between 13.4 and 15.7 eV) than was found for P (15.1–16.3 eV) or silicon (14.3–17.6 eV). Considering that at our beam energy, the lowest end of these energies should dominate the cross section of any elastic scattering process limited by momentum-conservation [55], we expected Al impurities to be highly mobile under electron irradiation. Instead, we found that of the seven Al–C_3_ sites we observed at atomic resolution for extended periods of time (at least 5 min), only the heteroatom shown in figure 2 migrated within the lattice. Notably, this occurred at significantly higher irradiation doses than has been previously reported for P or Si [29] (for example, Si could be expected to have jumped dozens of times during the ∼13 min series covered by the figure). The ejection of a C neighbor was observed five times, but twice the resulting Al–C_4_ site healed back to Al–C_3_. Further studies collecting statistically robust data at multiple primary beam energies beyond our scope here will need to be performed to understand this discrepancy.

#### 2.1.3. Aluminium–nitrogen substitutions

Aluminium, with its three valence electrons, could be expected to form strong covalent bonds with nitrogen, which has five. Al–N co-doping has been theoretically considered, and was proposed to stabilize the three-coordinated Al site [49] forming an Al–C_2_N configuration (with one of the three C neighbors being substituted by N). Indeed, N co-dopants, presumably sputtered from the SiN TEM-grids during the Ar plasma irradiation, are occasionally found at Al sites in our samples, creating not only previously considered three-coordinated but also four-coordinated Al–C_3_N configurations.

Analysis of our experimental image intensities (figure 3) reveals that Al dopants in both three- and four-coordinated configurations can have one neighbouring atom with a higher-than-carbon MAADF intensity, i.e. an element with a higher atomic number [56]. These atoms were confirmed as N using EELS, with a characteristic *K*-edge core-loss spectrum of N recorded from a Al–C_2_N site shown in figure 3(c).

The experimentally determined projected Al–N distances were 1.51 ± 0.06 Å (Al–C_2_N) and 1.94 ± 0.08 Å (Al–C_3_N), slightly shorter than those calculated from relaxed DFT models (1.58 and 1.98 Å, corresponding to bond lengths of 1.81 and 1.98 Å, respectively). Notably, the projected Al–N distance in the three-coordinated Al–C_2_N site is more than 0.1 Å longer than the Al–C distance despite only a 0.05 Å difference in the bond length, indicative of the out-of-plane distortion of the site, while in the nearly atomically flat four-coordinated site, the differences are negligible. In contrast to the purely carbon-containing Al–C_4_ site, the out-of-plane displacement of the C bonding atoms in the Al–C_3_N site are present only one the side of the defect that does not contain N. The binding energies of the Al dopants into the nitrogen-containing sites were −4.88 eV (Al–C_2_N) and −7.13 eV (Al–C_3_N), and thus while N co-doping is energetically somewhat unfavorable for the three-coordinated configuration (+1.1 eV), it is found to stabilize the four-coordinated site by as much as −2.4 eV.

#### 2.1.4. N–Al bond inversion

**Figure 4.**
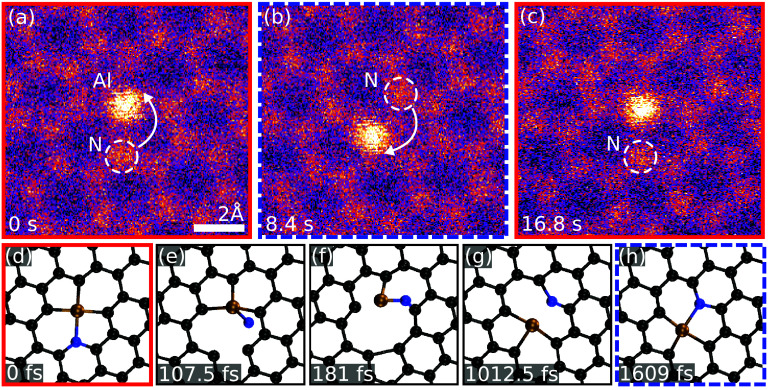
Rotation of the Al–C_3_N site (STEM/MAADF images and DFT/MD snapshots). (a) Initial configuration. The arrow marks the movement of the N atom from the first frame (a) to the second (b). The model images below (d)–(f) show the structure with C (black), N (blue) and Al (brown) atoms. In the second frame (b), the Al has moved downwards, while the N is now upwards from it. The structure still comprises of two pentagons and two hexagons, with no atoms lost, but Al and N are bound to different C atoms apart for the left-side neighbour of Al. The arrow marks the change with respect to the next frame (c), where the site is back to its initial configuration. (d) Relaxed atomic model of Al–C_3_N corresponding to (a). (e) Transfer of 16.5 eV of kinetic energy at a slight angle with respect to the plane normal displaces the N after 108 fs, rotating it out of the plane over the Al. (f) After 181 fs the N, still bound to the Al, also binds to a C upwards from the Al. (g) The Al relaxes after 1012 fs into a configuration where it is bound to three C, while the N is momentarily bound to only two C after the Al has shifted. (h) After 1609 fs, the Al–N bond is reestablished. This configuration corresponds to the intermediate frame in (b), after which the process can (by symmetry) reverse with a new electron impact and restore the configuration to the original one (frame in (c) and model in (d)).

We also observed non-destructive dynamics for Al–C_3_N: during imaging, the Al–N bond rotates, as shown in figures 4(a)–(c). We could find only one single-step process that can explain the observed outcome, which was confirmed by our DFT/MD simulations (see figures 4(d)–(h)): energy is imparted from a probe electrons to the N atom, displacing it out of the plane so that it subsequently passes over the Al atom while remaining bound to it, followed by the Al relaxing into the vacancy left by the N. A configuration that is by symmetry equivalent to the starting point is thus reached, but the site has rotated and shifted. A similar process has been predicted for the Fe–C_4_ defect [57]. In our experiment, the site changed back into its original configuration (figure 4(c)) after several seconds of further irradiation.

In the DFT/MD simulation shown in figures 4(d)–(h), this process was modelled by imparting a kinetic energy of 16.5 eV on the N atom with the initial momentum vector having a polar angle of 20 degrees from the out-of-plane *z* axis and an azimuthal angle of 20 degrees from the *x* axis (aligned along the armchair lattice direction of graphene). The same outcome was achieved also for azimuthal angles of 10 and 45 degrees, but could notably not be found in our simulations for the Al–C_4_ site, which does not contain nitrogen. Although the required kinetic energy exceeds the maximum transferable energy from a 60 keV electron in the static approximation of the elastic knock-on process, lattice vibrations can increase this energy [58, 59] and electronic excitations may in turn lower the threshold [60, 61].

### 2.2. Discussion

Notably, knock-out events of the Al heteroatom itself were rare in our experiments, limited to a single one from an Al–C_2_N configuration. Taking into account the total accumulated dose on all imaged Al dopants (which had several distinct local environments, so that this rough estimate should be strictly taken as an upper bound), this results in a displacement cross-section of 10^−3^ barn. Further, unlike has been observed for Si [38], Ge [23] and P [29], we did not observe any instances of Al being replaced by C during our experiments.

We expect that grain boundaries are highly reactive, and thus both creation of vacancies and incorporation of Al atoms is enhanced there. However, they are also typically covered by contamination that is particularly difficult to remove, and we did not purposefully try to find any in our specimen. The dynamical processes under electron irradiation very likely would not take place in the same way if the local lattice configuration is not perfectly hexagonal.

Our study is complementary to the recent work of Ullah *et al* [44], though there are notable differences. Although potentially easier to scale up, their chemical synthesis route necessitates the transfer of the sample from the Cu growth substrate, while our post-synthesis physical route can be applied to any free-standing graphene sample. However, they were able to achieve a higher concentration of Al, and we did not observe defects with multiple dopants. On the other hand, they detected a high concentration of oxygen in their samples, including oxidized Al nanoparticle byproducts, whereas our samples have never been exposed to ambient and our Al nanoparticles are correspondingly completely metallic (as confirmed by EELS).

Finally, although Ullah *et al* [44] certainly did substitute Al into the graphene lattice, their EEL spectra were not collected on single atoms and thus cannot spectrally distinguish different local Al–C coordinations. Further, comparing our atomically resolved spectra (figures 1(g) and (h)) with their areal acquisition, we believe that some of the shown spectral response is due to Si impurities, which their chemically insensitive high-resolution TEM imaging cannot differentiate from Al (indeed, this is challenging even with annular dark-field STEM). Thus, our results are complementary to theirs and the availability of both synthesis routes is a welcome development that is set to open up Al-doped graphene to a broad range of studies.

## 3. Conclusion

We substituted Al dopants into free-standing graphene via intermittent mono- and divacancies created using argon ions and filling these with aluminium from physical vapour deposition. The substituted sites were observed without ambient exposure using atomically resolved imaging and spectroscopy. The Al were found in three- and four-coordinated configurations, bound either to three or four C atoms, but occasionally also to N dopants. Different configurations of Al dopants and Al–N dopant-pairs showed dynamical behaviour induced by the electron irradiation at a 60 keV primary beam energy. The theoretically predicted direct exchange process that has been found to enable atomically precise atom manipulation of covalently bound impurities was experimentally confirmed also for three-coordinated Al dopants, but was found to be clearly slower than expected from earlier simulations. Electron irradiation of four-coordinated Al–N double-heteroatom sites was observed to lead to a new kind of non-destructive dynamical process where the Al site rotates around one C neighbor due to beam-induced out-of-plane dynamics of the N. Our findings thus increase the tools at our disposal for engineering the properties of graphene at the atomic scale.

## 4. Materials and methods

### 4.1. Sample preparation

A monolayer graphene sample was prepared from commercially available CVD-grown graphene (Graphenea ‘Easy Transfer’). The graphene, covered with a sacrificial polymer layer and originally 1 × 1 cm^2^ in size, was cut with a surgical blade into a size slightly larger than the TEM grid. This was floated in a beaker filled with deionized water, and then fished out onto a perforated SiN TEM grid (Ted Pella, hole diameter of 2.5 *µ*m) held with tweezers. After transfer, the sample was baked overnight under 10 mbar Ar/H^2^ atmosphere (95/5 molar ratio) at 450 ^∘^C to remove the polymer layer, leaving regions of free-standing graphene. SWCNTs were dry-deposited on the graphene surface to reduce mechanical oscillations emerging in ultra-clean samples [50]. The sample was baked overnight in vacuum at nominal 180 ^∘^C temperature prior to introducing it into the interconnected near-UHV system (base pressure 10^−8^ mbar), where heteroatom substitution and characterization were undertaken.

### 4.2. Heteroatom substitution

The surface of the free-standing graphene was cleaned [51] on a *µ*m-scale from ubiquitous hydrocarbon contamination using a *in-situ* laser diode-pumped solid-state laser (473 nm, Cobolt Blues^™^ 25, Cobolt AB) with added focusing optics (750 *µ*s laser pulse, power 25 mW and spot size ca. 560 *µ*m^2^). Amorphous contamination was thereby either evaporated or accumulated at reactive areas, such as around the graphene-SWCNT interface. The pre-cleaned specimen was then transferred in near-UHV to a chamber containing a plasma generator and a diode heating laser [51].

Low-energy Ar^+^ ions from the plasma generator with a current of 16.5 nA (exposure time 300 s) and anode and extractor voltages both at 0 V were used to irradiate the graphene. The measured ion energy for these parameters is approximately normally distributed with a mean of ca. 170 eV and a standard deviation of ca. 30 eV. The ion irradiation corresponds to a dose equivalent to ca. 3 × 10^13^ cm^−2^. During the Ar plasma irradiation, the sample was simultaneously irradiated with ca. 100 mW of laser power spread to a spot size of ca. 0.3 × 1.5 mm^2^ to elevate its temperature and to mitigate hydrocarbon buildup.

The plasma treatment was followed by thermal evaporation of an Al target (evaporation slug, 99.999%, Sigma-Aldrich) heated to 955 ^∘^C, producing an Al partial pressure of 10^−8^ mbar (the chamber base pressure was 5 × 10^−10^ mbar). The total evaporation time was 20 s, resulting in ∼5 nm diameter Al clusters covering large portions of the specimen, as well as single Al dopants in the graphene lattice.

### 4.3. Microscopy and spectroscopy

STEM medium-angle annular dark-field (MAADF) images were acquired with a Nion UltraSTEM 100 (probe convergence semiangle 30 mrad, detector semiangular range 60–200 mrad). EELS was carried out in the same instrument with a Gatan PEELS 666 spectrometer fitted with an Andor iXon 897 electron-multiplying charge-coupled device camera [22]. To estimate the beam current, we calibrated the current of electrons impinging on the virtual objective aperture, which is recorded when images are taken, against the beam current measured on the drift tube of the EELS. We used a 60 keV primary beam energy with a beam current of ca. 50 pA.

### 4.4. Density functional theory

DFT simulations were carried out with the grid-based projector-augmented wave (GPAW) software package [62] using the Perdew–Burke–Ernzerhof (PBE) functional [63]. Each site was placed in a 6 × 6 hexagonal supercell of graphene with periodic boundary conditions (10 Å of vacuum in the perpendicular direction between the images) and a 5 × 5 × 1 **k**-point mesh, and both the cells and the atomic structures were relaxed [64] until maximum Hellman-Feynman forces were below 0.02 eV Å^−1^. The simulation scripts can be found as Supplementary Materials (available online at stacks.iop.org/TDM/9/035009/mmedia).

For the structural optimization and energetics, a plane-wave basis with a cutoff energy of 500 eV was used. Binding energies of the Al dopants were estimated by comparing the total energies of the relaxed structures to equivalent configurations where the Al atom was removed and the structure relaxed. In GPAW, the total energy of an isolated atom in vacuum is by definition zero, and thus this energy difference corresponds to the energy gained by the system when the Al atom is bound to the defect.

To further study the electron-beam-induced dynamics of the sites, MD simulations were conducted with a *dzp*-basis in the LCAO mode [65] with a grid spacing of 0.2 Å. A Velocity-Verlet timestep of 0.5 fs and a total simulation time of 1.5 ps were used. Each run started from an initial kinetic energy kick assigned to a selected atom whose magnitude was increased until a threshold energy value was found as a change in the atomic arrangement during the trajectory (as described in detail in previous work [48, 66]), and whose direction was optionally varied from the perpendicular direction [67].

EEL spectra of the Al substitutions were simulated with the CASTEP package [68] based on DFT with pseudopotentials generated on-the-fly. The structures were re-optimized using the PBE functional with a plane-wave cutoff energy of 500 eV and 3 × 3 × 1 **k**-point mesh until the forces on the atoms were below 0.04 eV Å^−1^. The single-atom EELS simulation of the $L_{2,3}$ response covers the transitions from the 2*p* core state of Al to 3204 unoccupied bands of the crystal without an explicit core hole included [69]. The final spectrum was broadened by the OptaDOS package [70] with adaptive broadening [71] using 1.0 eV Gaussian and 1.3 eV Lorentzian components.

## Data Availability

Open data, including scanning transmission electron microscopy image series and density functional theory relaxed structures and molecular dynamics trajectories, can be obtained from the University of Vienna repository Phaidra (https://doi.org/10.25365/phaidra.334).
